# Freezing at the His Bundle

**DOI:** 10.1016/s0972-6292(16)30815-4

**Published:** 2014-12-15

**Authors:** Kairav P Vakil, Venkat N Tholakanahalli

**Affiliations:** Division of Cardiology and Cardiac Electrophysiology, Department of Medicine, University of Minnesota and Veterans Affairs Medical Center, Minneapolis, Minnesota, USA

**Keywords:** His bundle, cryoablation

"Our greatest weakness lies in giving up. The most certain way to succeed is always to try just one more time." - **Thomas A. Edison**

Prior to 1980, the only method to treat cardiac arrhythmias besides anti-arrhythmic drug therapy was open-heart surgery, that involved either dissecting or freezing the arrhythmic focus with a hand-held metal probe. Soon thereafter, early reports of percutaneously performed cardiac ablation were published that marked the beginning of a new era in the field of cardiac electrophysiology (EP). As our understanding of EP and ablation physics continues to evolve, remarkable advancements have been made over the last 30 years in ablation technology - moving from direct-current energy in the early 1980s, to radio-frequency (RF) ablation, to the more recent cryoablation in the last decade.

Accessory pathways (AP) are the commonest reasons for supraventricular tachycardia in the pediatric population. Antero-septal, mid-septal, and para-Hisian AP locations are the least common locations in Wolff-Parkinson-White syndrome [1-3]. RF ablation has been commonly used to treat these APs. Although highly efficacious, septal AP ablation with RF carries a high risk of heart block (previously reported to be as high as a 5%) [1,4,5] due close anatomic proximity of these APs to the atrio-ventricular (AV) node and the His bundle. As such, the need for a permanent pacemaker is a dreaded complication of this procedure, especially in the pediatric population. Cryoablation is an emerging technology that has shown to be much safer in this regard for ablating septal APs [6,7].

In this issue of the *Journal*, Liberman and colleagues studied 70 patients who underwent cryoablation for antero-septal APs. Six of these patients were identified to have a His-electrogram on the cryo catheter at the site of successful AP ablation [8]. All 6 patients had evidence of ventricular pre-excitation. Using 4-6 mm tip cryoablation catheters the authors report a 100% acute procedural success rate, though disease recurrence was noted in 2 patients within 30-days that necessitated a second ablation procedure. While intra-procedural transient AV block and A-H prolongation occurred in two patients, no patients had high-grade heart block during a median follow-up of 13 months. The authors concluded that antero-septal APs can be safely and successfully ablated using cryo energy even when a His bundle electrogram is recorded from the tip of the ablation catheter.

The authors must be commended for conducting and reporting such a study as it would be highly unusual for electrophysiologists to deliver ablation lesions at an area where a discrete His-electrogram can be identified - unless intended for the purpose of intentional AV nodal ablation. While the findings are interesting, a few limitations must be addressed. First, it is difficult to ascertain whether the His signal noted at the ablation site was a near- or far-field electrogram. Given that cryo lesions are focused and shallow, it is likely that presence of a low frequency/ far field His signal may have been protective. Second, 'slants' have also been described in antero-septal APs [9]. Differential pacing form right atrial appendage versus lateral coronary sinus, may unfold other alternative sites, probably away from the His location, with an AP potential that would be good ablation targets. Third is the sample size of six patients.

Nevertheless, these findings advance our knowledge and complement the findings previously published. Prior reports have consistently shown that cryoablation for substrate targets that are in close proximity to the AV node, is associated with a significantly lower incidence of heart block as compared to traditional RF ablation [10]. In a large systematic review [11], Atienza and colleagues reported the procedural results of 3,775 patients who underwent septal AP ablation using either cryo or RF energy. While the incidence of complete heart block was 2.8% with RF, there were no cases of persistent AV nodal injury with cryoablation.

Several speculations can be made as to why cryoablation may be safer in this regard. First, cryoablation has the advantage of helping to localize the site of an AP using the technique of 'cryo-mapping' [6] in which selected sites are cooled to a temperature that reversibly and temporarily halts local electrical activity, but not to a temperature low enough to cause permanent tissue damage [12,13] While it does not appear that cryo-mapping was performed in these 6 patients; this strategy should be routinely employed during such high-risk ablations. Second, once a critical freezing temperature is reached, the cryo catheters do not move in reference to cardiac and respiratory motion unlike RF catheters. It is important to remember that APs are, generally, very small discrete structures. Although cryo and RF lesions create lesions of equal depth, the endocardial surface affected by cryo is smaller due to catheter stability, yielding a smaller volume of ablated tissue [14]. Cryo lesions are, therefore, more 'focused' and ideal for targeting small APs without injury to nearby structures. Lastly, application of RF around the AV node frequently provokes junctional rhythm that can make the assessment of antegrade AV conduction during ablation slightly challenging. The other method of checking AV node integrity is by assessment of retrograde VA conduction. In such cases situations however, even when RF application is halted with first signs of retrograde VA block, severe heart block may occur and usually persist. During cryoablation it is uncommon to see junctional rhythm [6,13], and thus antegrade AV conduction can be easily monitored with atrial pacing during ablation as done in the present study by Liberman et al.

Despite the beneficial safety profile, procedural success rates with cryoablation remain suboptimal as compared to RF [11,15]. Even in the present study 2/6 (33%) patients had early disease recurrence requiring a redo ablation. However, in young patients who require perinodal AP ablation, cryoablation with cryo-mapping can be used as a reasonable initial strategy, especially when one is willing to accept a lower rate of procedural success to minimize the risk of procedural AV block. If unsuccessful, RF ablation can be pursued. As far as ablating an AP at a site of the His electrogram is concerned, studies with larger sample sizes are required to assess procedural safety and future implementation of such a strategy into clinical practice.
